# A hybrid deep image prior and compressed sensing reconstruction method for highly accelerated 3D coronary magnetic resonance angiography

**DOI:** 10.3389/fcvm.2024.1408351

**Published:** 2024-09-12

**Authors:** Zhihao Xue, Sicheng Zhu, Fan Yang, Juan Gao, Hao Peng, Chao Zou, Hang Jin, Chenxi Hu

**Affiliations:** ^1^ National Engineering Research Center of Advanced Magnetic Resonance Technologies for Diagnosis and Therapy, School of Biomedical Engineering, Shanghai Jiao Tong University, Shanghai, China; ^2^ Shenzhen Institutes of Advanced Technology, Chinese Academy of Sciences, Shenzhen, Guangdong, China; ^3^ Department of Radiology, Zhongshan Hospital, Fudan University and Shanghai Medical Imaging Institute, Shanghai, China

**Keywords:** coronary magnetic resonance angiography, deep image prior, compressed sensing, unsupervised learning, image reconstruction

## Abstract

**Introduction:**

High-resolution whole-heart coronary magnetic resonance angiography (CMRA) often suffers from unreasonably long scan times, rendering imaging acceleration highly desirable. Traditional reconstruction methods used in CMRA rely on either hand-crafted priors or supervised learning models. Although the latter often yield superior reconstruction quality, they require a large amount of training data and memory resources, and may encounter generalization issues when dealing with out-of-distribution datasets.

**Methods:**

To address these challenges, we introduce an unsupervised reconstruction method that combines deep image prior (DIP) with compressed sensing (CS) to accelerate 3D CMRA. This method incorporates a slice-by-slice DIP reconstruction and 3D total variation (TV) regularization, enabling high-quality reconstruction under a significant acceleration while enforcing continuity in the slice direction. We evaluated our method by comparing it to iterative SENSE, CS-TV, CS-wavelet, and other DIP-based variants, using both retrospectively and prospectively undersampled datasets.

**Results:**

The results demonstrate the superiority of our 3D DIP-CS approach, which improved the reconstruction accuracy relative to the other approaches across both datasets. Ablation studies further reveal the benefits of combining DIP with 3D TV regularization, which leads to significant improvements of image quality over pure DIP-based methods. Evaluation of vessel sharpness and image quality scores shows that DIP-CS improves the quality of reformatted coronary arteries.

**Discussion:**

The proposed method enables scan-specific reconstruction of high-quality 3D CMRA from a five-minute acquisition, without relying on fully-sampled training data or placing a heavy burden on memory resources.

## 1 Introduction

Three-dimensional coronary magnetic resonance angiography (CMRA) is a promising imaging modality for the assessment of coronary artery disease (CAD) due to its non-invasiveness and freedom from ionizing radiation (1–3). Despite these benefits, CMRA still suffers from a long scan time, because of the need to achieve millimeter-level 3D whole-heart imaging. To address this issue, highly undersampled acquisition is often employed to accelerate CMRA, with acceleration factors ranging from 7 to 10 (4). These high acceleration rates can cause serious degradations of image quality due to the incurrence of aliasing artifacts and noise amplification in the image. Therefore, reconstruction algorithms that minimize these artifacts and noise at high acceleration rates are highly desirable.

Various reconstruction methods have been developed to improve image quality for accelerated MRI, and some have been applied to the CMRA reconstruction. Early methods are often based on the compressed sensing theory. These methods leverage general image properties to accelerate imaging, such as sparsity in the wavelet domain (5), finite total variation (6), or low-rankness of signals extracted from non-local patches (7). Some of these methods have achieved reconstructions of adequate quality with a scan time of 5–7 min (4, 8, 9). Recently, learning-based methods have emerged as a new genre of reconstruction methods for fast MRI. These methods can learn image priors from existing data and have shown the state-of-the-art performance in various reconstruction tasks. Among these methods, some are purely data-driven, such as AUTOMAP (10) and U-Net (11), while others are partly model-driven, which include deep unrolling (12–15), plug-and-play (16, 17), and methods based on learned explicit priors (18, 19). Usually, model-driven methods are more robust and generalizable than purely data-driven methods. However, despite an improved generalizability for the model-driven methods, a major challenge remains: they often require a substantial amount of training data, which can be scarce or unavailable in various scenarios, including 3D CMRA (20). Additionally, performance degradation due to distribution shifts between training and testing datasets is not negligible. For instance, several studies have found performance degradation of deep learning-based methods in reconstructions of out-of-distribution data (21, 22). Finally, certain methods, such as deep unrolling, are challenging to use in 3D imaging due to the high memory demand of network training. Given these limitations of supervised methods, there is a growing demand for database-free, self-supervised, or unsupervised reconstruction approaches.

Deep imaging prior (DIP) (23) has emerged as a powerful unsupervised technique for solving inverse problems in image processing, including denoising, super-resolution, and inpainting. DIP does not need external training data; instead, DIP optimizes the parameters of a randomly initialized deep neural network from scratch for each reconstruction. During training, the network learns to map a vector of noise to the reconstructed image, whose regularization is attributed to the inductive bias captured by the network architecture. Recently, several DIP-based methods have been proposed for MRI reconstruction, exhibiting an improvement of image quality when compared with other unsupervised methods (24–29). Compared with supervised methods, DIP does not require any training data, reduces the memory requirement compared with deep unrolling, and induces less concern on its generalizability under different undersampling patterns or anatomies (24, 25). Recently, the application of DIP has been extended to cardiac imaging, including cardiac magnetic resonance fingerprinting (MRF) (27), functional CMR (28), and 2D + *t* dynamic MRI (30). Some research has also investigated the combination of DIP and classical regularization methods within an iterative optimization framework (31, 32).

Although DIP has been used in 2D imaging, no prior study has utilized DIP for 3D CMRA reconstruction. Thus, it remains unclear whether 2D DIP can generalize well to 3D reconstruction in terms of reconstruction quality and memory usage. In this work, we fill this gap by proposing a novel hybrid DIP-CS method for 3D CMRA reconstruction. This approach combines the unsupervised DIP model and the total variation (TV) regularization within a framework that uses the alternating direction method of multipliers (ADMM). We compared the proposed 3D DIP-CS method with several alternative methods, including iterative SENSE, CS-wavelet, CS-TV, 2D slice-by-slice DIP, and 2D slice-by-slice DIP equipped with 2D TV regularization with both retrospective and prospective undersampling experiments.

## 2 Theory and methods

### 2.1 Compressed sensing MRI and deep image prior

The reconstruction of 3D MRI can be considered as an inverse problem in the following format:

(1) m=Af+ϵ=DFSf+ϵ

Where $f\;\in C^{N}$ is the 3D image, $m\;\in C^{M}$ is the 3D k-space measurement, ϵ is the noise, and A is the matrix representation of the forward imaging model. In parallel imaging, the forward model consists of three linear operators: coil sensitivity encoding S, the Fourier transform operator F, and k-space undersampling operator D. Due to the undersampling, the inverse problem is ill-conditioned. Consequently, regularization is usually required to solve the problem. Many approaches based on compressed sensing theory leverage the sparsity of images in a certain transform domain to improve the conditioning. The objective function of reconstruction is described as follows in Equation 2:

(2) $$\underset{f}{\mathrm{argmin}}\|m-Af{\|}_{2}^{2}+\lambda \Phi (f)$$

where $\left\|m-{Af}_{2}^{2}\right\|$ is the data-fidelity term, Φ(f) the regularization term, and *λ* the regularization weight. Several algorithms like ADMM can be used to solve Equation 2.

Deep image prior (23) is an approach proposed to solve image recovery problems, such as image inpainting, denoising, and reconstruction. In general DIP schemes, a random but fixed latent noise variable $z\;\in R^{L}$ serves as the input to an un-trained deep convolutional neural network (CNN) G(z;θ), where $\theta \in R^{d}$ are the parameters of the network, which are either randomly initialized or tuned with pre-training. During training, θ is optimized to make the network output G(z;θ) minimize the reconstruction loss with respect to the measurement m through the model A:

(3) $$\underset{\theta }{\theta^{*}=\mathrm{argmin}\;}\|m-AG(z;\theta ){\|}_{2}^{2}$$

Where $\theta^{*}$ is the optimized network weights. No prior information is needed to solve Equation 3; instead, the inductive bias of the convolutional neural network can implicitly enforce a natural appearance in the reconstructed images (30). Typically, an over-parameterized network *G* is used (33), which means that the number of network parameters *d* is much larger than the output dimension *N*.

### 2.2 Proposed framework

In this work, we propose a hybrid DIP-CS framework for 3D image reconstruction. Because k-space is fully sampled in the readout direction of MR acquisition, we firstly perform 1D inverse Fourier transform in this direction to partition k-space into a sequence $\left\{m^{\left(i\right)}\right\}$, where $m^{\left(i\right)}$ is the k-space data for the *i*th slice along the readout direction. We then solve the 3D reconstruction problem by a slice-by-slice application of regular 2D DIP. However, a slice-by-slice reconstruction not only may induce inter-slice inconsistency of image intensities, but also lacks regularization power over the inter-slice direction. To address this issue, we combine 2D DIP with a 3D TV regularization, which is separately applied along the intra-slice dimension and the inter-slice dimension. The objective function is shown below:

(4) $$\underset{\theta }{\mathrm{argmin}}\overset{N_{slice}}{\underset{i=1}{\sum }}(\|m^{\left(i\right)}-AG(z^{\left(i\right)};\theta^{\left(i\right)}){\|}_{2}^{2}+\lambda \|G(z^{\left(i\right)};\theta^{\left(i\right)}){\|}_{TV_{yz}})+\lambda \|G(z;\theta ){\|}_{TV_{x}}\;\;\;$$

where $z^{\left(i\right)}$ and $\theta^{\left(i\right)}$ are the corresponding latent representation and network weights of each slice, G(z;θ) is the 3D image obtained by concatenating all the network output slices, and $\|\cdot {\|}_{TV_{yz}}$ and $\|\cdot {\|}_{TV_{x}}$ are the intra-slice and inter-slice TV regularization, respectively. To minimize this function, we combine the ADMM with the proposed DIP-CS formulation by using variable splitting. The problem is transformed into a constrained optimization as in Equation 5:

$$\underset{\theta }{\mathrm{argmin}}\overset{N_{slice}}{\underset{i=1}{\sum }}(\|m^{\left(i\right)}-AG(z^{\left(i\right)};\theta^{\left(i\right)}){\|}_{2}^{2}+\lambda \|G(z^{\left(i\right)};\theta^{\left(i\right)}){\|}_{TV_{yz}})+\lambda \|v{\|}_{TV_{x}}\;\;\;\;$$

(5) $$s.t.\;\;v^{\left(i\right)}=G(z^{\left(i\right)};\theta^{\left(i\right)})$$

**Figure 1:**
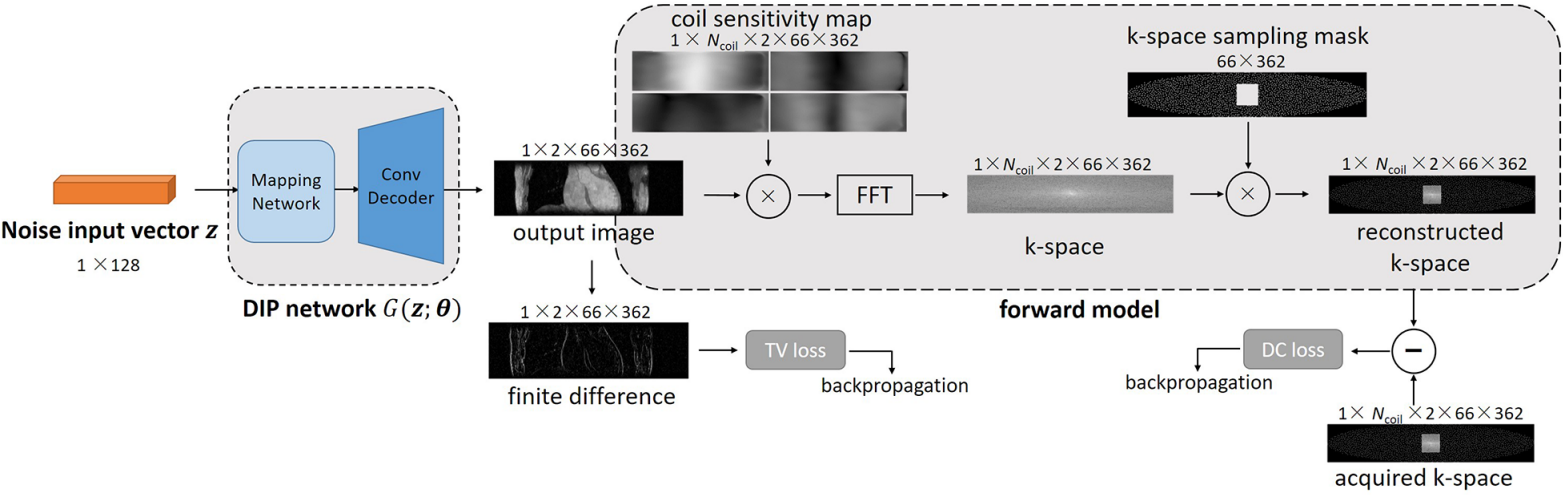
Overview of the proposed DIP-CS method for 3D CMRA reconstruction. The input of each slice is a noise vector that remains fixed during optimization. To maintain inter-slice continuity of the reconstructed image, the noise vectors stay in a straight-line manifold across different slices. The reconstruction network in DIP-CS is a ConvDecoder with a fully-connected mapping network. The loss for updating the network parameters consists of two parts: the data-consistency loss and total-variation loss. The data-consistency loss is the *l*2 loss between k-space of the network output and undersampled k-space data. The total-variation loss is calculated from the output image over both intra-slice and inter-slice directions.

Where v is the auxiliary variable used to replace G(z;θ) in the inter-slice regularization term. Figure 1 shows an overview of 3D DIP-CS. The numbers denote the size of each object in this framework. z is a vector of length 128. We solve Equation 5 based on the ADMM algorithm, which recursively executes Equations 6a–c:

(6a) $$\theta_{t+1}=\underset{\theta }{\mathrm{argmin}}\overset{N_{slice}}{\underset{i=1}{\sum }}(\|m^{\left(i\right)}-AG\left(z^{\left(i\right)};\theta^{\left(i\right)}\right){\|}_{2}^{2}+\lambda \|G\left(z^{\left(i\right)};\theta^{\left(i\right)}\right){\|}_{TV_{yz}}\;+\frac{\rho }{2}\|G\left(z^{\left(i\right)};\theta^{\left(i\right)}\right)-v_{t}^{\left(i\right)}+u_{t}^{\left(i\right)}{\|}_{2}^{2})\;\;$$

(6b) $$v_{t+1}=\underset{v}{\mathrm{argmin}}\lambda \|v{\|}_{TV_{x}}\;+\frac{\rho }{2}\|G(z;\theta_{t+1})-v+u_{t}{\|}_{2}^{2}\;\;\;$$

(6c) $$u_{t+1}=u_{t}+G(z;\theta_{t+1})-v_{t+1}$$

Note that the first subproblem in Equation 6a is solved using DIP with a 2D TV regularization slice-by-slice, whereas the second subproblem in Equation 6b is solved along the inter-slice direction only, which can be computed in parallel. Furthermore, since adjacent slices should have similar appearances, we hypothesize that $\left\{z^{\left(i\right)}\right\}$ across different slices should reside in a connected manifold in the latent space. We thus add an additional constraint on $\left\{z^{\left(i\right)}\right\}$, as described in Equation 7, which asks $\left\{z^{\left(i\right)}\right\}$ to reside in a learnable straight-line manifold:

(7) $$z^{\left(i\right)}=(1-\alpha^{\left(i\right)})z^{\left(0\right)}+\alpha^{\left(i\right)}z^{\left(N_{slice}-1\right)},\;\alpha^{\left(i\right)}=i/(N_{slice}-1)$$

Where $z^{\left(0\right)}$ and $z^{\left(N_{slice}-1\right)}$ are sampled from the standard uniform distribution U(0,1), and $\alpha^{\left(i\right)}$ controls the position on this straight line. This constraint was inspired by a previous work of DIP (30), which constrains the latent representation of dynamic MRI to a learnable straight-line manifold.

**Figure 2:**
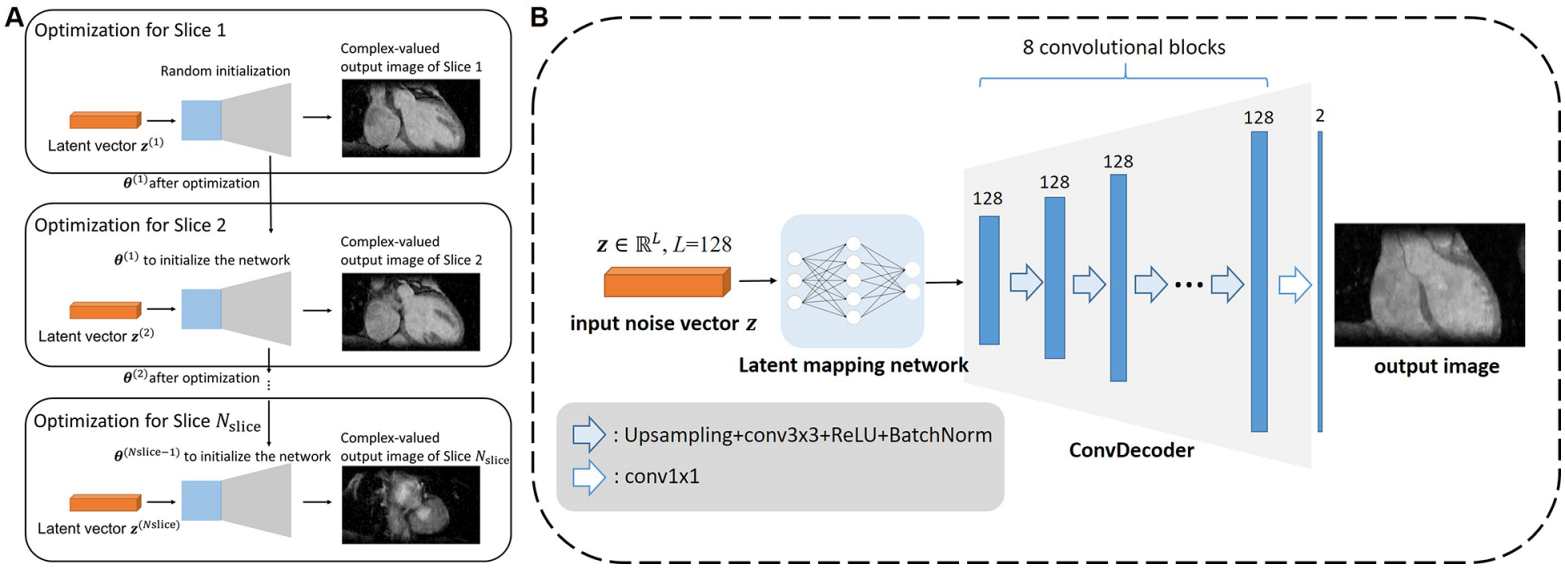
Architecture of the proposed framework. **(A)** The diagram for how the multi-slice reconstruction was performed using the network and multi-slice latent vectors. For each slice except the first, the network parameters were initialized with those obtained from the previous slice to expedite the reconstruction process. **(B)** Network architecture adopted in the proposed 3D DIP-CS framework. The latent mapping network comprises of fully-connect layers that map the random noise to the input of the ConvDecoder. The ConvDecoder comprises of 8 network blocks, each formed by an up-sampling layer, a convolutional layer, and a batch normalization layer. The output is an image with two channels holding the real and imaginary parts.

**Figure 3:**
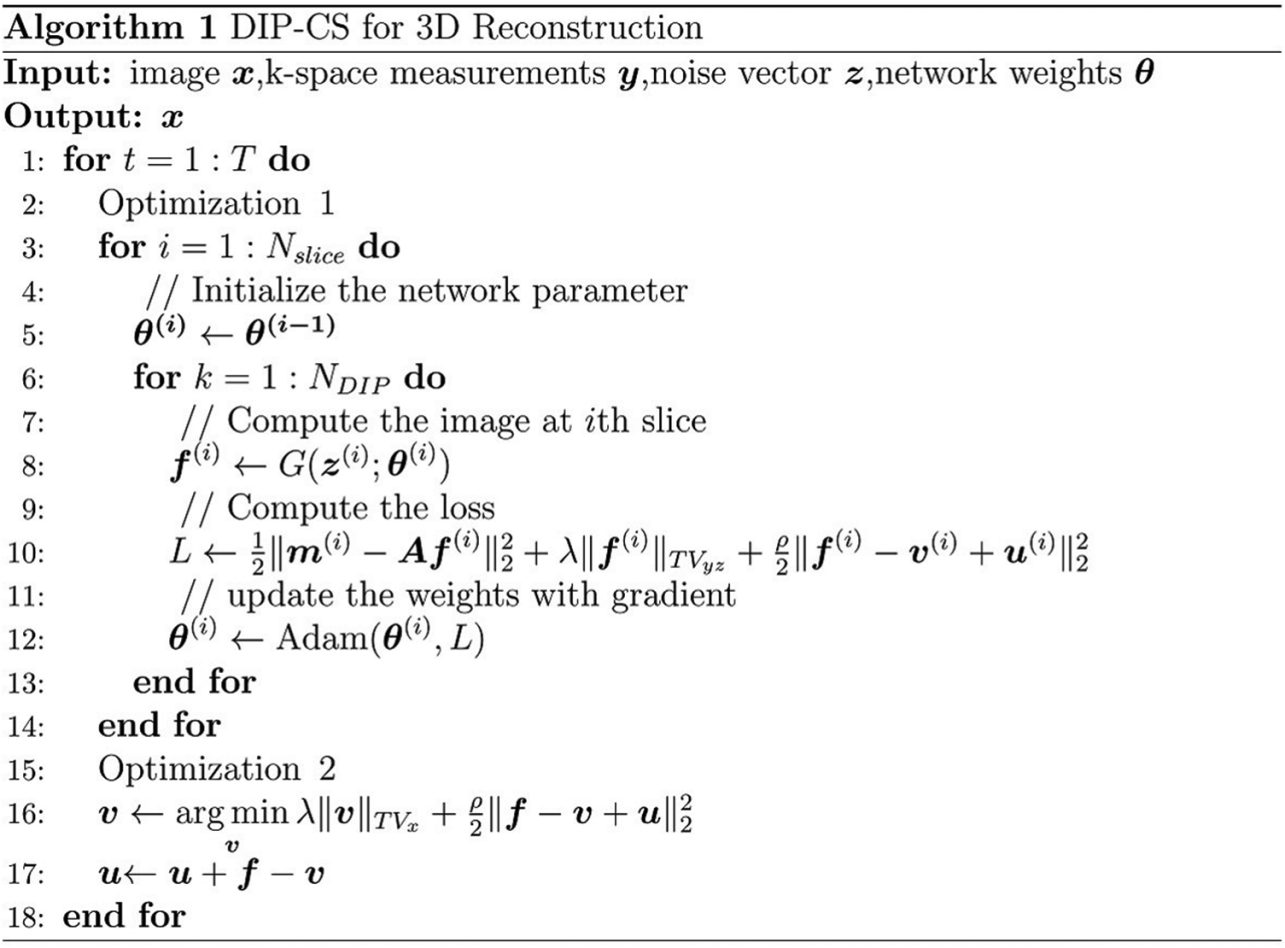
The DIP-CS algorithm for 3D reconstruction.

To accelerate the reconstruction of each slice, we propose initializing the network weights of the *i*th slice based on those of the (*i-*1)th slice, as shown in Figure 2A. The whole algorithm is summarized in Algorithm 1 of Figure 3. Optimization in Equation 6a was performed for *N*_DIP_ times and the whole iterative process of Equations 6a–c was *T* iterations.

### 2.3 Network architecture

Many network architectures can be selected as the backbone of the un-trained model, such as CNN in the original DIP work (23), ConvDecoder (25), MLP in DeepDecoder (34, 35), skip-connected U-Net (36), and even Transformer (26, 37). In this paper, we modified the architecture in ConvDecoder (25) to construct the neural network $G(z^{\left(i\right)};\theta^{\left(i\right)})$ in DIP-CS. The network architecture to model each image slice is illustrated in Figure 2B. The input to the network is a fixed vector with length *L*, containing uniformly distributed noise. Similar to the MapNet in 2D + *t* time-dependent DIP (30) and the “Mapper” in zero-Shot Learned Adversarial Transformer (37), we firstly used a mapping network consisting of two fully-connected layers to map the noise vector into the input space of the modified ConvDecoder. The first layer in the mapping network outputs a 128-channel vector, and the second transforms this vector into a 128-channel 16 × 16 feature map. The ConvDecoder module then produces the output image by progressively increasing the image resolution across layers. The input and output of ConvDecoder have two channels for real and imaginary parts because of the complex-valued MR images. Each block in this module comprises of a nearest-neighbor up-sampling layer, a 3 × 3 128-channel convolutional layer, ReLU function and a batch normalization layer as in the original work. The final layer only uses a 1 × 1 convolution to linearly combine the channels.

## 3 Experiments

### 3.1 Datasets preparation

We conducted both retrospective and prospective experiments to investigate the effectiveness of the proposed DIP-CS method. For the retrospective experiment, 10 healthy volunteers were recruited and underwent CMRA imaging in a 3.0 T MRI scanner (uMR 790, United Imaging Healthcare Co. Ltd., Shanghai, China) with a 12-channel torso coil and 32-channel spine coil. Written informed consent was obtained from all subjects before the scan. No contrast agent was used in this study. A navigator-gated, electrocardiogram-triggered, 2-point Dixon T2-prepared spoiled gradient echo sequence was used for 3D CMRA (8, 38), which generated two images of different echo times for each subject. The acquisition window for each cardiac cycle was 164 ms, which was placed in the quiescent diastolic phase to reduce cardiac motion. A four-chamber cardiac cine imaging was performed prior to the CMRA scan to determine the subject-specific optimal trigger delay to minimize cardiac motion during the acquisition. Data were acquired with image readout in the anterior-posterior (AP) direction, and phase encoding in the left-right (LR) and feet-head (FH) directions. Scan parameters of the 3D GRE sequence with Cartesian sampling include: TE1/TE2 = 2.24/3.17 ms, TR = 5.21 ms, duration of T2 preparation = 32 ms, filed of view (FOV) = 400 × 300 × 90 mm^3^(LR × AP × FH), acquisition matrix = 362 × 272 × 60 (10% oversampling in FH direction), acquired resolution = 1.10 × 1.10 × 1.50 mm^3^, flip angle = 10°, bandwidth = 1,070 Hz/pixel, and T2-prep TE = 32 ms. Two-fold undersampling in the phase encoding direction *k_y_* was performed on the basis of elliptical scanning to shorten the scan time. Supplementary Figure 1A gives a demonstration of the undersampling trajectory. Coil compression was used to compress the multi-coil data into 12 channels (39) using the BART toolbox (40). The 24 × 24 central reference lines were fully-sampled and used for ESPIRiT (41) coil sensitivity estimation. The mean scan time was 23 min 36 s. The 3D image was then reconstructed using iterative SENSE with Tikhonov regularization (regularization weight = 0.1). The result was treated as the ground truth and used to generate retrospectively undersampled data. Eight-fold undersampling was used based on a pseudo-random poisson-disc mask in the *k*_y_-*k*_z_ plane (as in Supplementary Figure 1B). From the 2× accelerated acquisition to the 8× undersampling, the scan time can be reduced to approximately 5–6 min. Different reconstruction methods were then used to reconstruct the images from the retrospectively undersampled data.

For the prospective experiment, we scanned another 13 volunteers with a self-navigated, electrocardiogram-triggered, 2-point Dixon T2-prepared spoiled gradient echo sequence (38), which is different from the navigator-gated sequence used in the retrospective experiment. The sequence uses a variable-density Cartesian undersampling trajectory in the *k*_y_-*k*_z_ plane, where the acquisition in each heartbeat passes the k-space center. The k-space center sampling is then reconstructed by 1D inverse Fourier transform to provide a self-navigating signal for retrospective binning of the data. All data were binned into 4 respiratory phases, and data from the end-expiration bin were used for reconstruction. Data were acquired with image readout in the FH direction, and phase encoding in the LR and AP directions in this sequence. The sequence had the following imaging parameters: TE1/TE2 = 2.24/3.17 ms, TR = 5.21 ms, FOV = 400 × 300 × 132–150 mm^3^(RL × FH × AP), acquisition matrix = 362 × 272 × (88–100), flip angle = 10°, bandwidth = 1,070 Hz/pixel, and T2-prep TE = 32 ms. We scanned each volunteer for 5 min to obtain the undersampled data. To provide reference images as an evaluation of reconstruction quality, we continued the scan for another 15 min to obtain nearly fully-sampled data (R ≈ 2.5 for the end-expiration bin). The reference images were then reconstructed using iterative SENSE with Tikhonov regularization (regularization weight = 0.1).

### 3.2 Ablation study

We performed the ablation study to evaluate the effectiveness of 3D DIP-CS by comparing the method to (1) standard 2D DIP applied slice-by-slice, where the TV regularization was completely removed, and (2) 2D DIP-CS applied slice-by-slice, where only the inter-slice TV regularization was removed. We then assessed the performance of the three methods on the retrospective dataset.

### 3.3 Comparison methods

We applied four baseline methods for comparison to show the advantage of the proposed method. These methods included (1) zero-filling, which directly fills the unsampled k-space points with zero followed by the inverse Fourier transform and coil combination, (2) iterative SENSE, which is a classical reconstruction method for parallel imaging, (3) CS-TV, which uses total-variation regularization in the classic iterative CS-based reconstruction, and (4) CS-wavelet, which is a CS-based algorithm based on a *l*1-norm constraint on the wavelet transform of the image. The last 3 methods were also compared in multiple undersampling rates and patterns to investigate the generalizability.

### 3.4 Image and statistical analysis

In our experiments, the two-echo acquisitions were reconstructed separately as two 3D images. To quantitatively evaluate the performance of the reconstruction methods, the peak signal-to-noise ratio (PSNR), structural similarity index (SSIM), and normalized mean-squared error (NMSE) were calculated on each 3D image volume with respect to the reference image.

In addition to the generic reconstruction quality comparison, we also compared the coronary vessel sharpness and conspicuity between different methods. To evaluate vessel sharpness, we firstly performed the two-point Dixon water-fat separation (43) based on the method described in (44) to generate the water image. We then reformatted the water image using curved planar reformation based on 3D Slicer (Version 5.2.2) (42) to visualize the coronary arteries. After that, we computed sharpness of the left anterior descending artery (LAD) and the right coronary artery (RCA) based on the intensity profile of vessel edges, using the method previously described in Ref (45, 46). To evaluate the coronary artery conspicuity, we invited a clinician (with more than 5 years of experience in cardiology) and a MR physicist (with more than 10 years of experience) to rate the images of reformatted coronary arteries. The images of different comparison methods were randomized, and their identity was blinded to the readers. For each image, the visualized vessel was given a rank using a five-point scoring system: score 1, uninterpretable (vessels that were almost indiscernible); score 2, poor (vessels that were visible but highly degraded by noise or artifacts); score 3, fair (vessels with moderately blurred borders); score 4, good (clear vessels but with slight blurring); score 5, excellent (vessels with sharply defined borders and structure). The average score from the two readers was used as the final score.

In tables, statistics for quantitative metrics were provided as mean ± standard deviation across all the subjects. Statistical significance of the difference between two methods was assessed by using the Wilcoxon signed-rank test (for expert scoring) and paired *t*-test (for others). Statistical significance of the difference between multiple methods was assessed using repeated-measures one-way analysis of variance (ANOVA) with Bonferroni correction. A *P* value less than 0.05 was considered statistically significant.

### 3.5 Implementation details

A platform equipped with 4 Intel Xeon Platinum 8260 (2.4 GHz) CPU and two NVIDIA A100 GPU was used for the experiments. Our framework was implemented with Pytorch 1.9.1 on Python 3.7.7. The proximal operator of total variation was solved using proxTV toolbox (47, 48). For each slice, the network of DIP was optimized for 1,000 iterations (*N*_DIP_ in Algorithm 1) using the Adam (49) optimizer with a learning rate of 5 × 10^−5^. After parameter tuning, the parameters in Algorithm 1 were set to be *ρ *= 0.001 and *λ *= 0.006. The number of outer loops was set to *T* = 3 for faster reconstruction. The results of optimizing the parameters were demonstrated in Supplementary Figure 2. The baseline CS-TV and CS-wavelet were implemented with BART (40). The regularization parameter was optimized on one sample and was finally set to be 0.01.

## 4 Results

### 4.1 Imaging with retrospective accelerations

#### 4.1.1 Comparison with other methods

**Figure 4:**
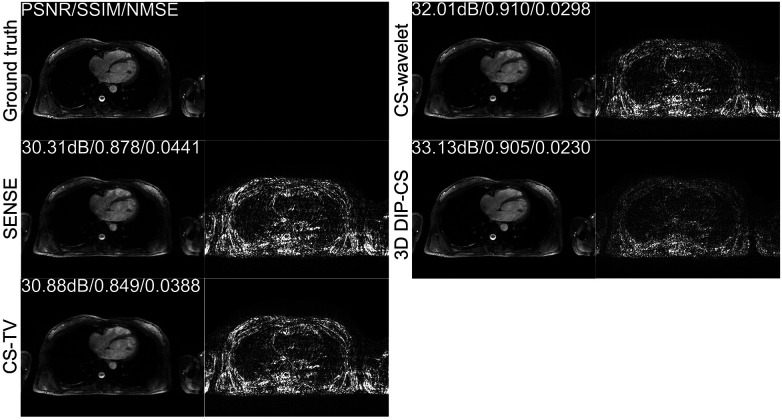
Reconstructions of CMRA in the transversal view from one representative subject, reconstructed with iterative SENSE, CS-TV, CS-wavelet, and the proposed 3D DIP-CS (R = 8). Corresponding error maps with respect to the fully-sampled ground truth are also shown. 3D DIP-CS led to improved reconstruction accuracy compared with these alternative methods.

**Figure 5:**
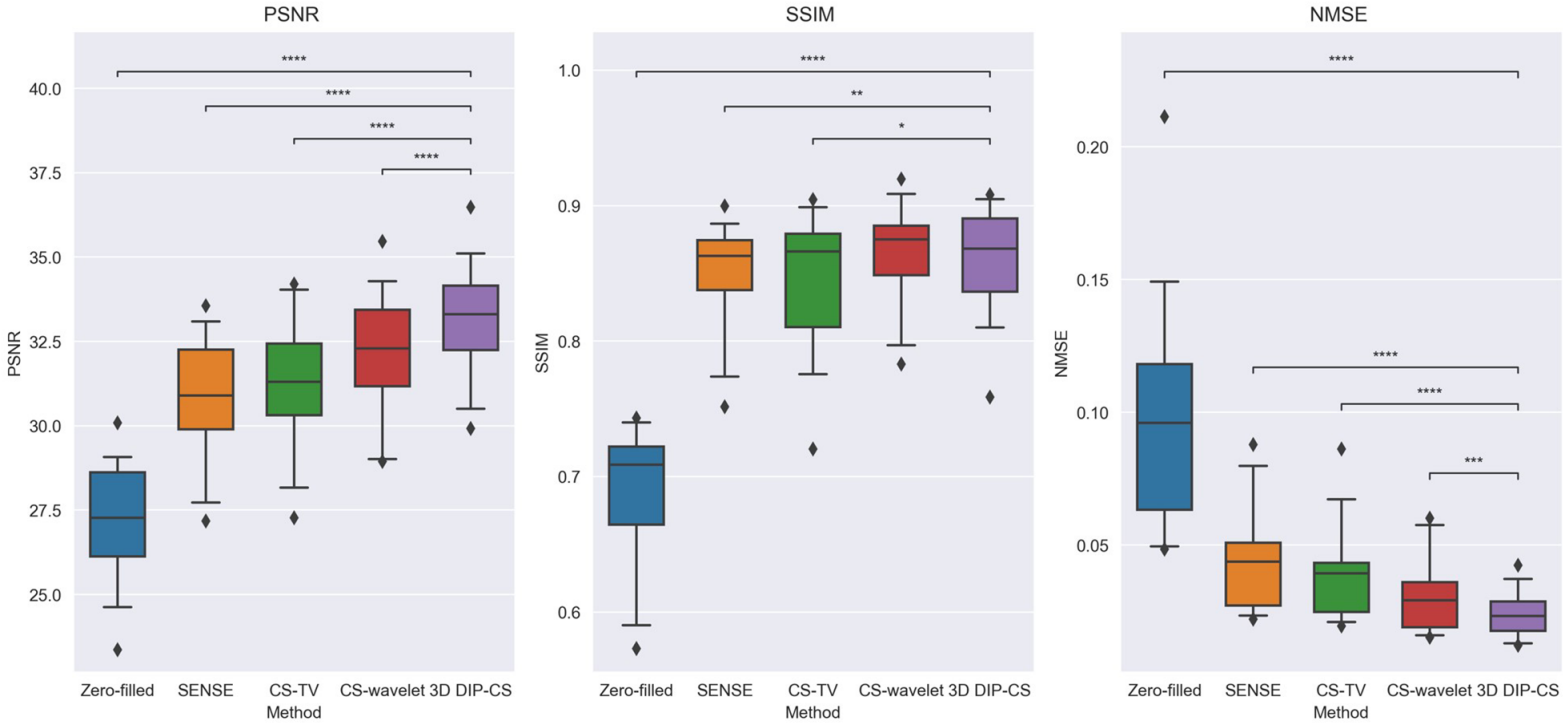
Image quality metrics of zero-filling, iterative SENSE, CS-TV, CS-wavelet, and 3D DIP-CS at R = 8. The 3D DIP-CS method outperformed other methods in terms of PSNR and NMSE. (*P < 0.05; **P < 0.01; ***P < 0.001, ****P < 0.0001).

Figure 4 shows the comparison of iterative SENSE, CS-TV, CS-wavelet, and the proposed method on R = 8. Compared with the other two methods, DIP-CS produced results with fewer artifacts in the image and lower reconstruction errors. The quantitative values calculated from the entire volume were also in accordance with the visual perception. Figure 5 shows the statistical comparison of these quantitative metrics across 10 subjects. The 3D DIP-CS method achieved higher PSNR and lower NMSE (PSNR: 33.20 ± 1.63 dB; NMSE: 0.0236 ± 0.0087) compared with SENSE (PSNR: 30.75 ± 1.75 dB, *P *= 5 × 10^−10^; NMSE: 0.0424 ± 0.0186, *P *= 2 × 10^−6^), CS-TV (PSNR: 31.22 ± 1.87 dB, *P *= 3 × 10^−6^; NMSE: 0.0382 ± 0.0174, *P *= 6 × 10^−5^), and CS-wavelet (PSNR: 32.25 ± 1.76 dB, *P *= 2 × 10^−5^; NMSE: 0.0299 ± 0.0128, *P *= 4 × 10^−4^). Differences in SSIM between DIP-CS and CS-wavelet were not statistically significant (SSIM: 0.862 ± 0.038 vs. 0.864 ± 0.038, *P *= 0.57) in our experiments.

#### 4.1.2 Performance on multiple acceleration factors

**Figure 6:**
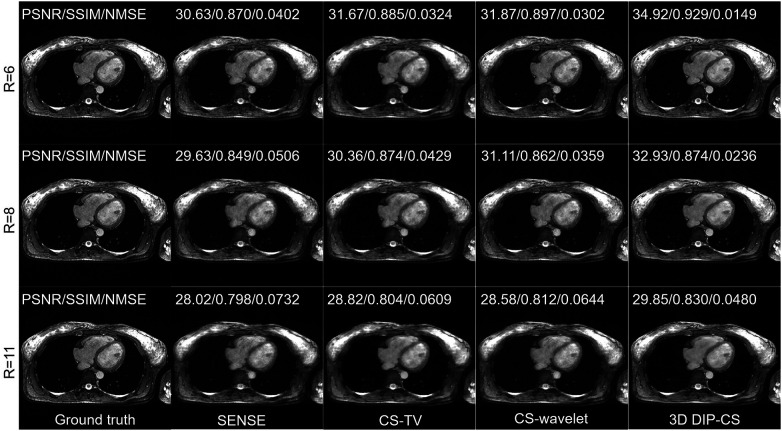
Reconstructions under different undersampling rates of R = 6, 8, and 11.

Figure 6 shows performance of the methods under different acceleration factors. We observed that under all the undersampling rates, 3D DIP-CS provided overall better reconstructions than the comparison methods. The generalizability to different acceleration factors is useful because the actual acceleration factor can be variant for a practical five-minute acquisition. Table 1 presents the quantitative results for reconstruction quality metrics using different undersampling rates. 3D DIP-CS led to overall better PSNR, SSIM, and NMSE than other methods.

**Table 1:** Average PSNR, SSIM, and NMSE for different methods across a variety of undersampling rates. Numbers indicate mean ± standard deviation.

Sampling rate	Method	PSNR(dB)	SSIM	NMSE
*R* = 6	SENSE	31.86 ± 1.88	0.872 ± 0.044	0.0331 ± 0.0155
	CS-TV	32.05 ± 1.92	0.881 ± 0.038	0.0317 ± 0.0142
	CS-wavelet	33.32 ± 2.01	0.901 ± 0.041	0.0238 ± 0.0116
	3D DIP-CS	**35.98 ** **± ** **1.89**	**0.928 **± **0.032**	**0.0127 **± **0.0054**
*R* = 8	SENSE	30.75 ± 1.75	0.848 ± 0.041	0.0424 ± 0.0186
	CS-TV	31.22 ± 1.87	0.847 ± 0.049	0.0382 ± 0.0174
	CS-wavelet	32.25 ± 1.76	**0.864 **± **0.038**	0.0299 ± 0.0128
	3D DIP-CS	**33.20 **± **1.63**	0.862 ± 0.038	**0.0236 **± **0.0087**
*R* = 11	SENSE	28.98 ± 1.78	0.789 ± 0.045	0.0639 ± 0.0287
	CS-TV	29.72 ± 1.61	0.0795 ± 0.051	0.0546 ± 0.0234
	CS-wavelet	29.58 ± 1.78	0.808 ± 0.044	0.0556 ± 0.0252
	3D DIP-CS	**30.83 **± **1.69**	**0.0824 **± **0.030**	**0.0408 **± **0.0149**

Bold value indicates the highest mean value of the metric among the comparison methods.

#### 4.1.3 Performance on the changed sampling pattern

**Figure 7:**
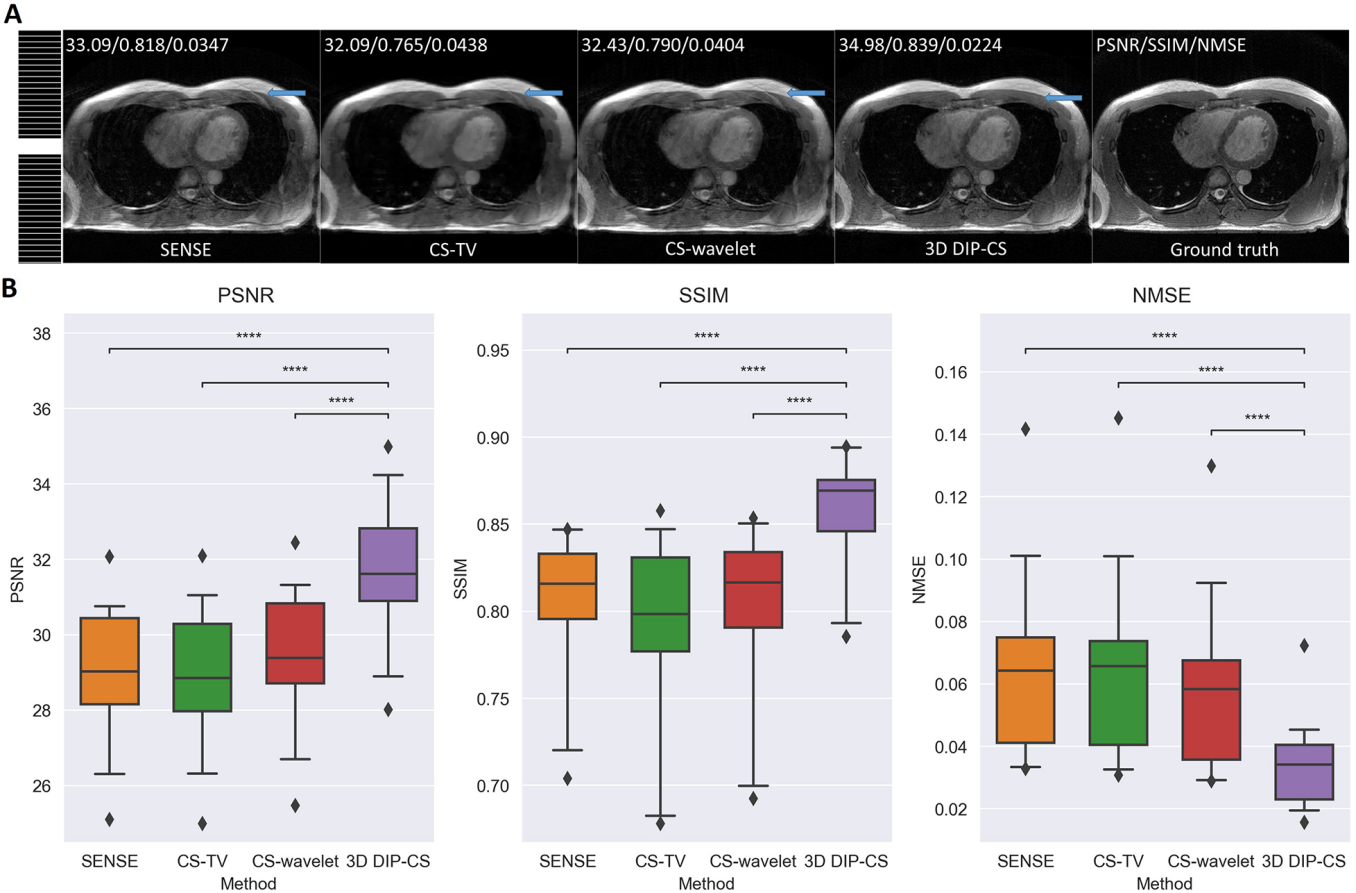
Reconstructed images under the uniform sampling pattern. **(A)** The sampling mask was shown on the left. 3D DIP-CS suppressed the aliasing artifacts better than the other methods, as pointed by the blue arrows. **(B)** Quantitative comparison of different methods under 1D uniform sampling. (****P < 0.0001).

To show the feasibility of the proposed method for uniform sampling, which is more commonly used in practice, we compared the reconstruction of each method with one-dimensional uniform undersampling. Figure 7A shows a representative result. In this example, only 3D DIP-CS successfully removed the aliasing artifacts of uniform undersampling, which can found in the reconstruction of other methods (blue arrows). Figure 7B shows the results of quantitative comparisons. 3D DIP-CS led to higher image qualities compared with other methods when uniform sampling was employed.

### 4.2 Ablation study

**Figure 8:**
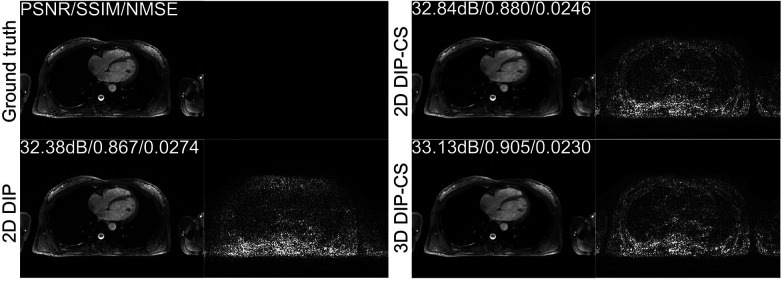
Reconstructions of CMRA and the error map in the transversal view using 2D DIP, 2D DIP-CS and the proposed 3D DIP-CS.

We employed ablation studies to investigate the effect of the intra-slice and inter-slice total variation regularization in the proposed 3D DIP-CS method. To do that, we compared the method with 2D DIP, which employed no total variation, and 2D DIP-CS, which employed only intra-slice total variation. Figure 8 shows the results. The proposed DIP-CS method achieved a better image quality compared with both 2D DIP and 2D DIP-CS. The 2D DIP method exhibited stronger noise in the heart region. While both 2D and 3D DIP-CS methods reduced noise, 3D DIP-CS yielded slightly higher values in image quality metrics, suggesting the addition of inter-slice CS further improved the reconstruction.

Table 2 shows the statistical comparison of different quantitative metrics between 2D DIP, 2D DIP-CS, and 3D DIP-CS. The proposed method achieved a higher PSNR/SSIM and lower NMSE (3D DIP-CS vs. 2D DIP: *P *= 5 × 10^−4^/0.004/0.019 for PSNR/SSIM/NMSE; 3D DIP-CS vs. 2D DIP-CS: *P *= 2 × 10^−7^/3 × 10^−4^/1 × 10^−6^ for PSNR/SSIM/NMSE) compared to the other methods. 2D DIP-CS also achieved a better PSNR and NMSE than 2D DIP (*P *= 0.003/0.037 for PSNR and NMSE), although SSIM was not significantly different between the two methods (*P *= 0.71).

**Table 2:** Comparisons of PSNR/SSIM/NMSE on 2D DIP, 2D DIP-CS, and 3D DIP-CS.

Method	PSNR (dB)	SSIM	NMSE
2D DIP	31.92 ± 1.97	0.850 ± 0.034	0.0344 ± 0.0248
2D DIP-CS	32.96 ± 1.63	0.852 ± 0.034	0.0249 ± 0.0091
3D DIP-CS	**33.20 ** **± ** **1.63**	**0.862 **± **0.038**	**0.0236 **± **0.0087**

Bold value indicates the highest mean value of the metric among the comparison methods.

### 4.3 Imaging with prospective accelerations

**Figure 9:**
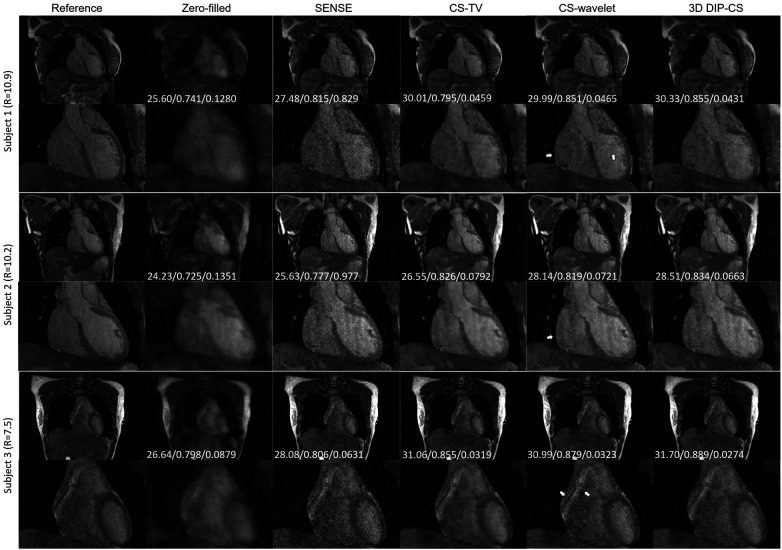
Reconstructions (in the coronal view) obtained using zero-filling, iterative SENSE, CS-TV, CS-wavelet and 3D DIP-CS from data acquired in five minutes. White arrows indicate the unsmooth details in the zoomed out images. PSNR, SSIM, and NMSE were given at the bottom of each image.

We assessed our method using real CMRA data acquired within 5 min, which had an acceleration factor of 7–11. An image reconstructed from a 15 min scan was used as the reference. Figure 9 shows a representative result. SENSE and CS-based methods were able to give reconstructions with reduced undersampling artifacts compared with zero-filling. However, noise appeared stronger in the SENSE reconstruction compared to the other methods. CS-TV significantly suppressed the noise but caused visible blurring in the images. CS-wavelet reconstructions mitigated some noise and undersampling artifacts; however, the images still showed some unsmooth features compared with the reference image (indicated by the white arrows in Figure 9). The 3D DIP-CS method removed most of the artifacts and well suppressed the noise, generating an image quality more similar to the reference image. Table 3 quantitatively compared the image quality metrics among these methods with respect to the 15 min reference images. DIP-CS achieved significantly higher PSNR, SSIM, and lower NMSE compared to classical CS-wavelet (PSNR: *P *= 0.001; SSIM: *P *= 0.015; NMSE: *P *= 0.001).

**Table 3:** Quantitative comparison of the reconstruction quality with respect to reference images among all the methods on the dataset.

Method	PSNR (dB)	SSIM	NMSE
Zero-filled	25.80 ± 0.96	0.738 ± 0.075	0.1161 ± 0.0315
SENSE	26.89 ± 0.97	0.768 ± 0.067	0.0889 ± 0.0194
CS-TV	29.16 ± 1.28	0.819 ± 0.044	0.0530 ± 0.0161
CS-wavelet	29.09 ± 1.10	0.826 ± 0.061	0.0564 ± 0.0166
DIP-CS	**29.82 ** **± ** **0.94**	**0.841 **± **0.050**	**0.0472 **± **0.0130**

Bold value indicates the highest mean value of the metric among the comparison methods.

### 4.4 Evaluation of the coronary arteries

**Figure 10:**
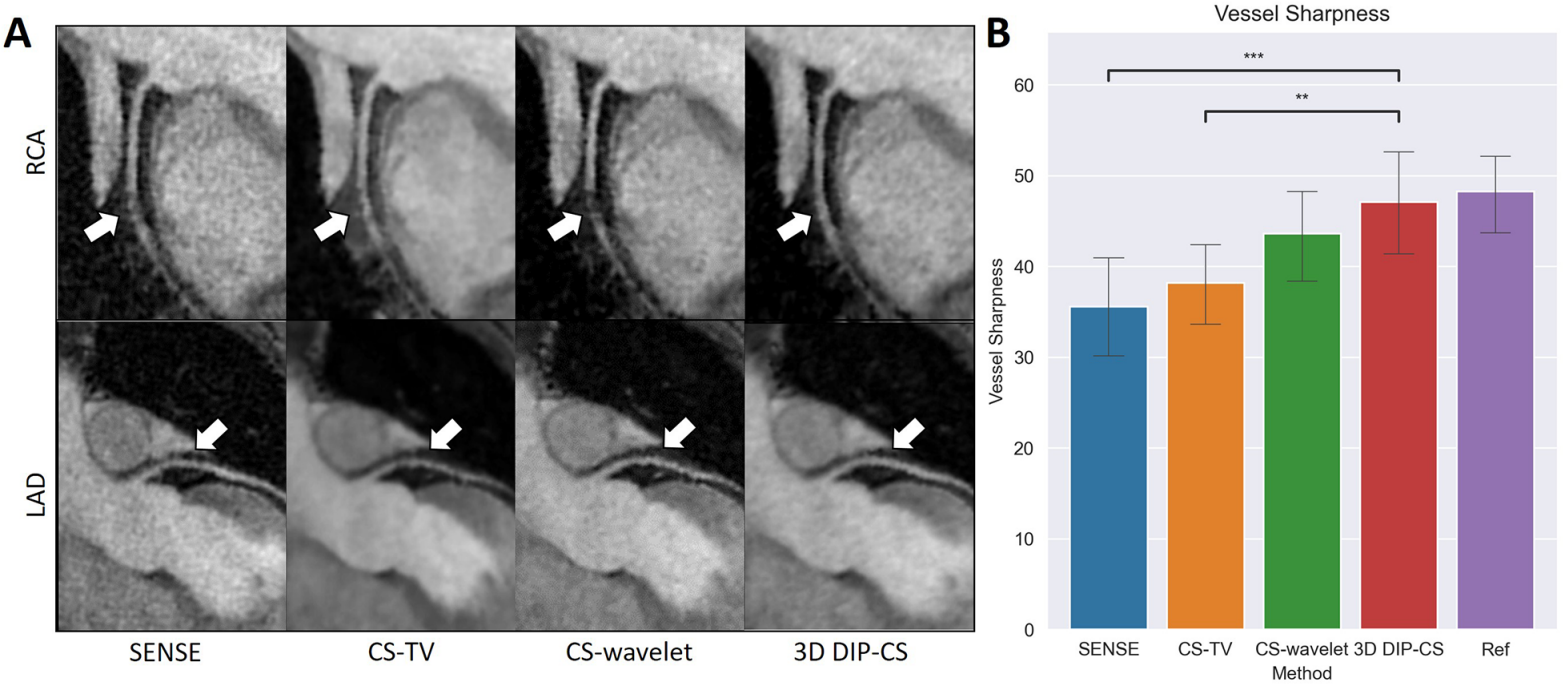
Visualized coronary arteries (RCA and LAD) from the reconstructions of SENSE, CS-wavelet, and 3D DIP-CS in a healthy subject. **(A)** The reformatted proximal segments of RCA and LAD in reconstructions of 3D DIP-CS showed better integrity and smoothness compared to other two methods (white arrows). **(B)** Quantitative metrics of vessel sharpness between different reconstruction methods. The sharpness metrics were compared between the four reconstruction methods and the fully-sampled reference image. Statistical comparison shows DIP-CS achieved higher vessel sharpness compared to the other methods. (*P < 0.05; ***P < 0.001, ****P < 0.0001).

Figure 10A compares reformatted coronary arteries of different methods using curved planar reformation. CMRA from SENSE reconstruction was noisy compared to that from CS-TV, CS-wavelet and DIP-CS. The boundaries of vessels in CS-TV reconstruction were slightly blurred. CS-wavelet reconstruction better mitigated the noise, yet residual artifacts and unsmooth edges of the coronary arteries were still visible. DIP-CS achieved the best coronary visualization among the compared methods.

**Figure 11:**
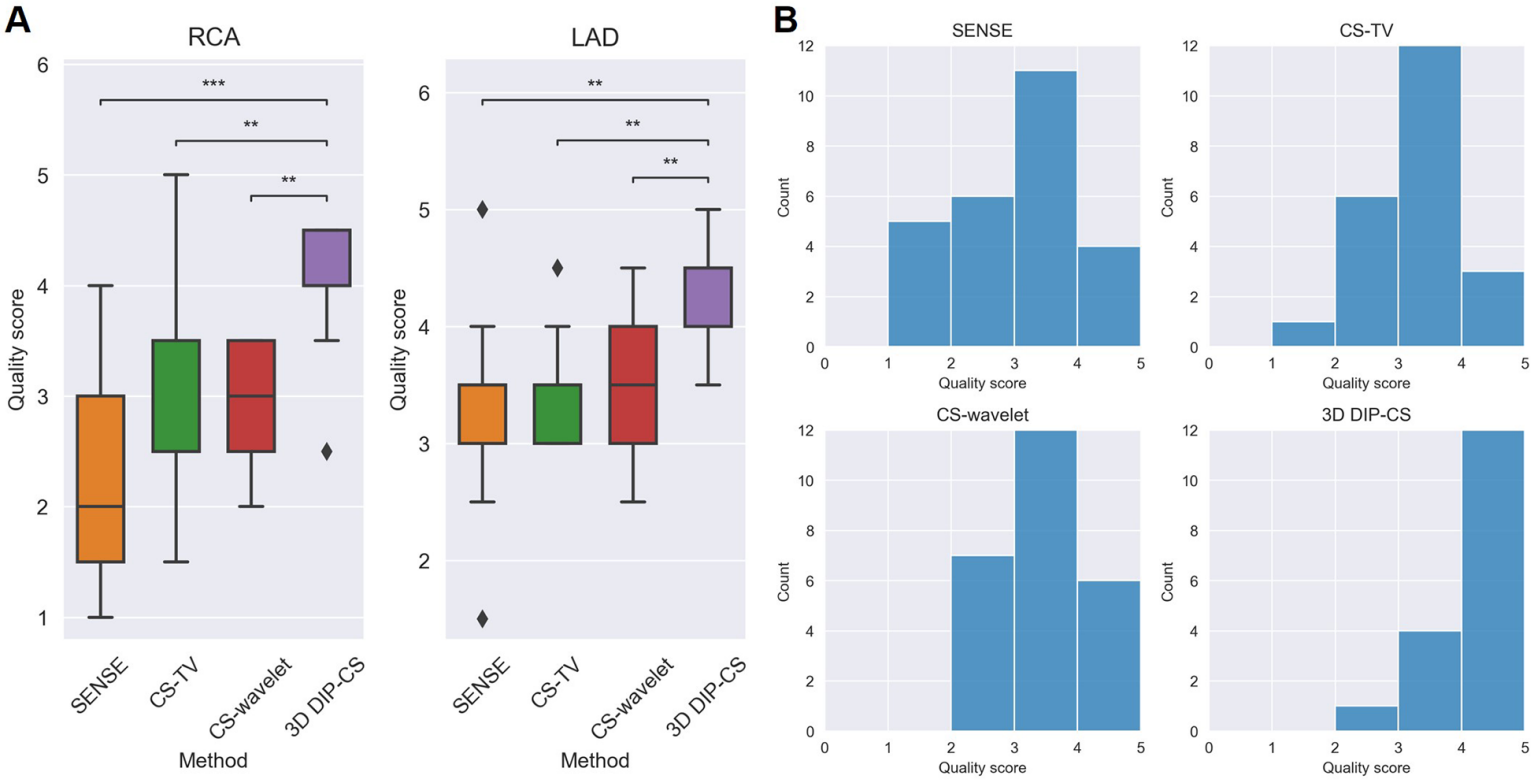
Qualitative scoring results for the healthy volunteers (*N *= 13) undergoing self-navigated CMRA scan with the proposed 3D DIP-CS and the comparison methods. A five-point scoring system was used to evaluate the quality of the reformatted vessels (5 = best). **(A)** Statistical comparison in RCA and LAD. **(B)** Distributions of scores in these methods. (**P < 0.01; ***P < 0.001).

Since reconstruction error metrics like NMSE and SSIM do not directly reflect the clinical quality of imaging, we compared the sharpness and conspicuity of the coronary arteries between different reconstruction methods. Figure 10B shows the results of quantitative vessel sharpness evaluation. 3D DIP-CS (47.2 ± 12.8%) significantly improved the vessel sharpness compared with CS-TV (38.1 ± 10.2%, *P *= 0.005 < 0.01), and SENSE (35.53 ± 12.5%, *P *= 9 × 10^−3 ^< 0.001). 3D DIP-CS also led to higher vessel sharpness compared with CS-wavelet (43.6 ± 10.9%, *P *= 0.20 > 0.05), although lacking statistical significance after Bonferroni correction. No significant difference was found between DIP-CS and the mildly undersampled reference images. Figure 11 shows the results of conspicuity scores rated by two readers. 3D DIP-CS resulted in significantly higher average score (RCA: 4.0 ± 0.6; LAD: 4.3 ± 0.5) than SENSE (RCA: 2.3 ± 0.9, *P *= 2 × 10^−4^; LAD: 3.3 ± 0.8; *P *= 0.007), CS-TV (RCA: 2.9 ± 0.9, *P *= 0.003; LAD: 3.4 ± 0.4, *P *= 0.003), and CS-wavelet (RCA: 3.0 ± 0.5, *P *= 0.002; LAD: 3.5 ± 0.6; *P *= 0.002).

## 5 Discussion

In this work, we introduce a scan-specific 3D DIP-CS method for accelerated CMRA reconstruction. The method demonstrates sufficient accuracy in reconstructing the 3D image from a 7-to-11-fold undersampling, which shortens the scan time of 3D CMRA from more than 20 min–5 min. Compared to the baseline methods such as iterative SENSE, CS-TV and CS-wavelet, our method exhibits superior mitigation of artifacts and noise, resulting in improved image quality for multiple k-space sampling rates and sampling patterns, and improved coronary artery sharpness and conspicuity. The short scan time underscores the potential of our method for clinical application.

Many existing learning-based reconstruction methods rely on large datasets for training. For example (50), trained a variational neural network on 2.7k images to perform nine-fold accelerated motion-compensated reconstruction (51). used data acquired from 45 subjects with an extensive data augmentation to train an unrolled MoCo-MoDL network for nine-fold accelerated CMRA. A recent study (52) used an unrolled CS-AI framework trained on 740k images of various anatomies and contrasts to accelerate data acquisition with an acceleration factor of 6 (the mean scan time was 8.1 min). Compared to these methods, our method has a key advantage in that it does not need external training data. Furthermore, since it is scan-specific, less concerns are present regarding its generalizability compared to fully-supervised methods (29). The method can thus have the potential to be used with different CMRA sequences and scan parameters, as shown in our experiments. Due to the same reason, it also holds promise for extension to solving other 3D reconstruction problems in MRI and other imaging modalities.

Compared with deep learning methods such as deep unrolling, our method requires significantly less GPU memory during optimization. In our experiment, 3D DIP-CS used less than 5 GB of GPU memory, making it a more practical and memory-efficient option. Owing to its efficient memory usage, the DIP-CS method could potentially be expanded with a 3D DIP network architecture, which has already been explored in a previous study for 3D PET imaging (53). This 3D DIP architecture is particularly useful for those 3D sampling trajectories that cannot be transformed into 2D slice-by-slice sampling, such as 3D radial sampling. In our initial validation, we have implemented such a 3D network architecture by changing the 2D convolutional layers to 3D layers. However, reconstruction results from the 3D DIP method were quite blurred, suggesting the presence of model underfitting. We opine that this lack of fitting may be due to the limited network capacity of a 3D DIP encoder compared to the large number of voxels, as previous studies have found that 2D DIP networks are often over-parameterized (20, 33, 54). Therefore, more sophisticated modifications may be needed to develop a 3D DIP method with high reconstruction quality.

**Figure 12:**
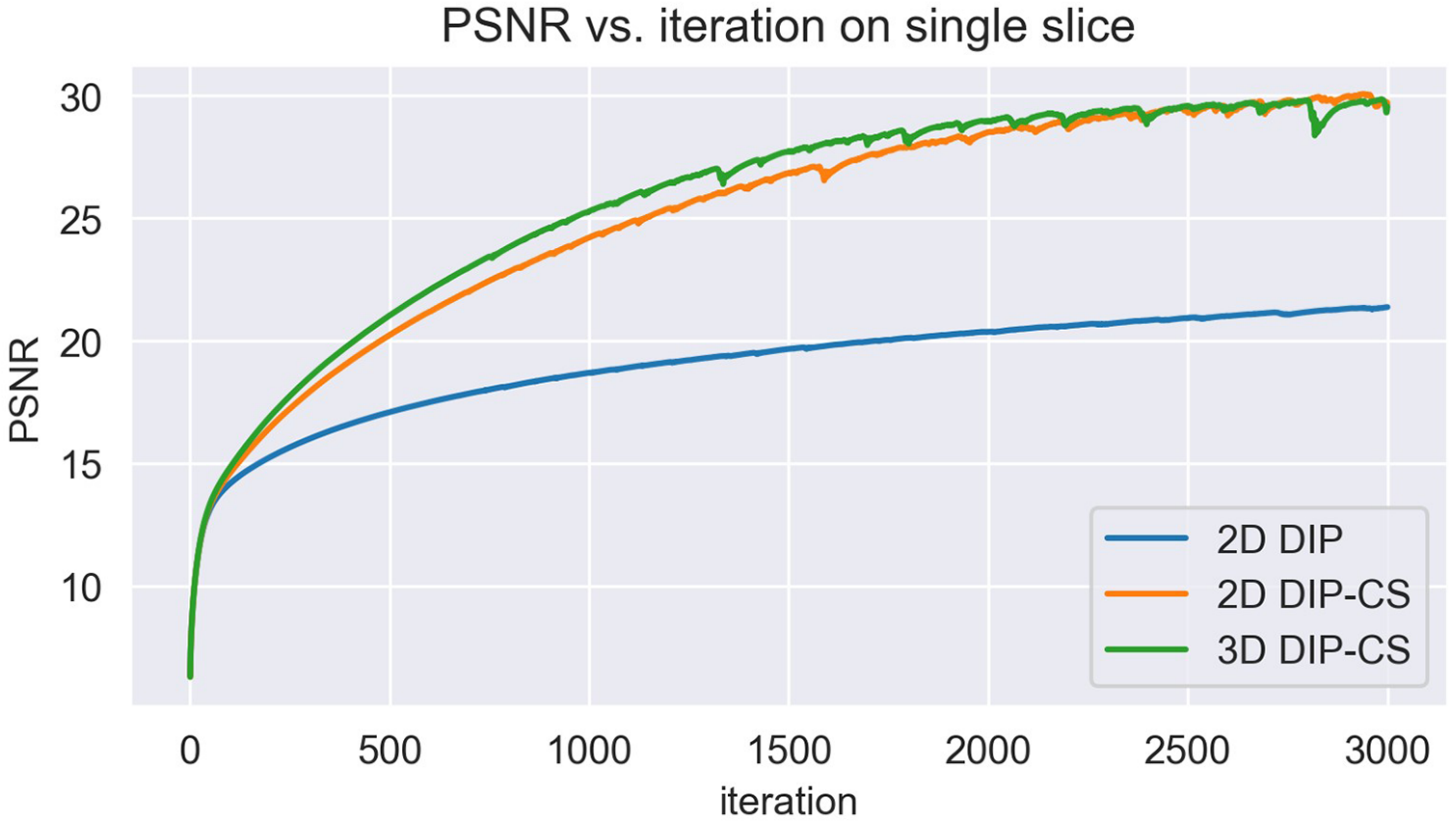
The variation of PSNR over each iteration of training. Initializations of network parameters were the same among the three methods for a fair comparison.

In this work, we compared the performance of 3D DIP-CS with several other unsupervised methods, including iterative SENSE, CS-TV and CS-wavelet. We found that 3D DIP-CS led to improved reconstruction accuracy and image quality. In addition to these benefits, we also found that the incorporation of TV regularization often resulted in faster convergence, with an example shown in Figure 12. This may be an additional advantage for a combination of DIP and the CS-based regularization.

The study design and the method have limitations. Firstly, due to the absence of a large CMRA dataset, our experiments were conducted on a relatively small dataset, which necessitates further studies on larger datasets. A larger number of subjects can enhance the statistical power of the analysis and increase reliability of the conclusions. Furthermore, a larger, multicenter dataset also contains more diverse data, providing a better vehicle for evaluating generalizability of the proposed method. Secondly, the acquisition window of our CMRA sequence was 164 ms, which was mildly longer than acquisition windows in previous studies [90–130 ms in (55)]. We will investigate the use of shorter acquisition window in future to reduce the potential adverse effect of cardiac motion. However, this limitation should not affect the relative differences between different reconstruction methods, as the same sequence was used for all of them. Thirdly, due to the limitation of computing resources and time, we only explored one type of network architecture. However, many other architectures such as Transformer or U-Net have been proposed as the backbone of DIP and may bring improvement to the results (26, 29, 37, 56). Finally, our method needs approximately 3 h to reconstruct a single volume, which needs acceleration before clinical translation. Long reconstruction time is a common issue for DIP. For example, in (24), the un-trained DIP-based method took 6 min for reconstruction of a single slice even after acceleration. Furthermore, due to the large data size, even classical CMRA reconstruction methods like non-rigid motion-compensated PROST need ∼50 min for reconstruction (9). Parallel computing and the use of 3D networks may be feasible approaches for further exploration.

## 6 Conclusion

In conclusion, we propose a hybrid DIP-CS method for the acceleration of 3D CMRA. By leveraging the implicit prior from the CNN architecture and the sparsifying prior from total variation, the proposed 3D DIP-CS method demonstrates the capability to recover images from 7 to 11 folds of acceleration without training on any external datasets. The superior image quality obtained by the proposed method renders it a useful method for accelerating 3D CMRA.

## Data Availability

The raw data supporting the conclusions of this article will be made available by the authors, without undue reservation.
