# Supplementary material for: A hybrid deep image prior and compressed sensing reconstruction method for highly accelerated 3D coronary magnetic resonance angiography
Source: Front Cardiovasc Med. 2024 Sep 12;11:1408351. doi: 10.3389/fcvm.2024.1408351 (PMC11424428; doi:10.3389/fcvm.2024.1408351)
Supplement: Supplementary file 1 [file Datasheet1.docx]

Supplementary Material of “A Hybrid Deep Image Prior and Compressed Sensing Reconstruction Method for Highly Accelerated 3D Coronary Magnetic Resonance Angiography”

# Supplementary Figures


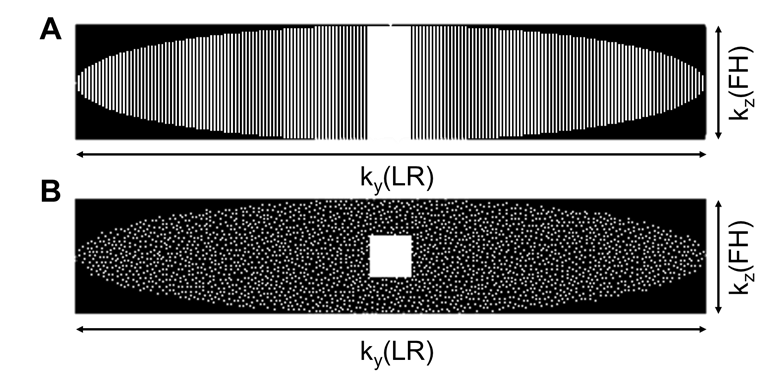


**Supplementary Figure 1.** The adopted Cartesian k-space sampling patterns in the study. (A) Two-fold undersampling in the phase encoding direction *k_y_* in data collection. (B) The eight-fold poisson-disc undersampling pattern in the *k_y_*-*k_z_* plane. (FH: feet-head; LR: left-right)


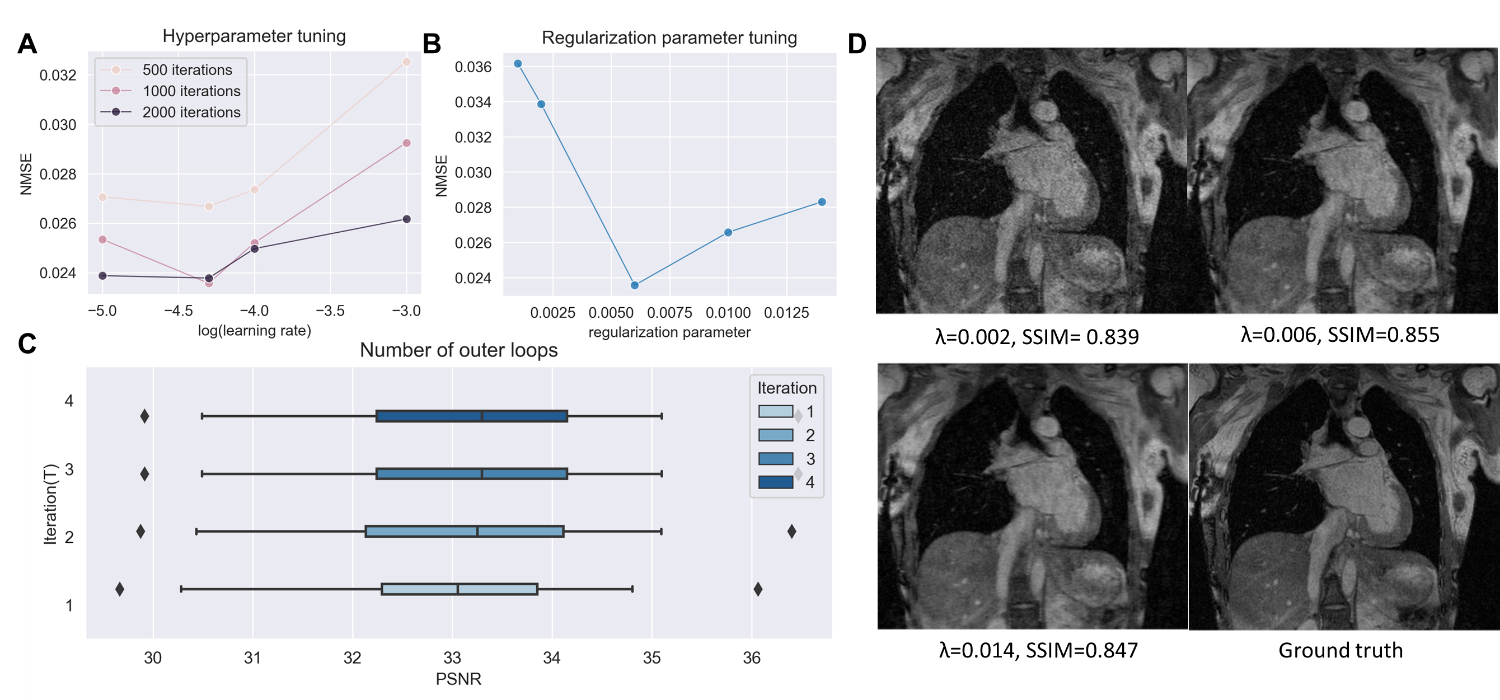


**Supplementary Figure 2.** The selection of the parameters in our algorithm. (A) The number of DIP iterations and learning rate were firstly chosen based on the validation sample. (B) The optimal regularization parameter was then selected. (C) The number of outer loops was determined as a trade-off of performance and time. (D) Images generated from different regularization weights. A very small weight results in large noise, whereas a very large weight causes blurring.
